# Longitudinal resting-state EEG-based modeling predicts phenoconversion and delineates heterogeneity in isolated REM sleep behavior disorder

**DOI:** 10.21203/rs.3.rs-9140218/v1

**Published:** 2026-03-26

**Authors:** Soonhyun Yook, Jung-Won Shin, Tae-Gon Noh, Gilsoon Park, Sumin Bae, Kang-Min Choi, Jung-Hwan Shin, Han-Joon Kim, Ki-Young Jung, Hosung Kim

**Affiliations:** University of Southern California; CHA University; Seoul National University; University of Southern California; Seoul National University Hospital, Seoul National University College of Medicine; Seoul National University of Science and Technology (Seoultech); Seoul National University Hospital, Seoul National University College of Medicine; Seoul National University Hospital, Seoul National University College of Medicine; Seoul National University Hospital, Seoul National University College of Medicine; University of Southern California

## Abstract

Isolated REM sleep behavior disorder is a high-risk prodromal syndrome for Lewy body diseases but highly heterogeneous. Neurophysiologic markers resolving this heterogeneity and predicting phenoconversion remain limited. We applied Subtype and Stage Inference (SuStaIn) modeling to longitudinal resting-state EEG from a prospective cohort of 285 participants. SuStaIn identified two subtypes: Subtype 1, a posterior beta–dominant, largely non-progressive phenotype associated with higher educational attainment and absence of phenoconversion, and Subtype 2, a frontal slow-wave–dominant phenotype characterized by progressive delta/theta increases and cognitive decline. Longitudinal validation demonstrated substantial subtype shifting in Subtype 1 and stage advancement predominantly in Subtype 2. In Kaplan–Meier analysis, subtype shifting and accelerated annual stage advancement (ΔStage ≥ 2) identified individuals at exceptionally high phenoconversion risk with survival approaching 0–15% by 7.5 years. These findings extend SuStaIn beyond cross-sectional inference by incorporating longitudinal subtype-shifting and stage-advancement features to stratify prodromal heterogeneity and predict neurodegenerative progression.

## Introduction

Isolated REM sleep behavior disorder (iRBD) is a parasomnia characterized by dream-enactment behaviors resulting from loss of normal REM sleep atonia.^1^ Beyond its sleep-specific manifestations, iRBD has emerged as one of the strongest clinical risk markers for prospective neurodegenerative disease, with longitudinal studies demonstrating high rates of phenoconversion to Parkinson’s disease (PD), dementia with Lewy bodies (DLB), and multiple system atrophy (MSA) over extended follow-up periods.^2,3^ Importantly, however, not all individuals with iRBD convert within a similar timeframe, and a substantial proportion remain clinically stable for many years,^2,4^ consistent with emerging evidence of biological variability in prodromal synucleinopathy, including differential progression of neuroinflammation reported in recent longitudinal imaging study.^5^ This heterogeneity highlights iRBD as a syndrome with divergent pathophysiological trajectories rather than a uniform prodromal stage.

Stratifying this heterogeneity is a central challenge for the field. While iRBD is widely regarded as a high-risk condition for Lewy body disorders at the population level, direct *in vivo* evidence of underlying pathology, such as α-synuclein aggregation, is limited. Consequently, there is a critical need for *biologically informed, non-invasive markers* that can distinguish subtypes and their trajectories within iRBD.

Accordingly, the field has shifted from modeling uniform neurodegeneration and estimating phenoconversion risk at the group level toward identifying disease progression patterns within heterogeneous prodromal populations. Such trajectory-based stratification improves prognostic precision by distinguishing low-risk from high-risk progressive pathways and by identifying individuals most likely to convert within a clinically actionable window.^6–10^ This is particularly relevant in Lewy body disease, where clinical expression depends on the timing and sequence of pathology across neural systems,^11^ and is supported by longitudinal iRBD cohorts and multicenter FDG-PET studies demonstrating non-uniform motor, non-motor, and metabolic progression.^9,10,12,13^ In parallel, numerous neurophysiological and neuroimaging biomarkers have been explored to identify progression patterns in iRBD, including dopaminergic imaging^14–16^, structural MRI^17,18^, and quantitative electroencephalography (EEG).^19–21^

Subtype and Stage Inference (SuStaIn) is a data-driven probabilistic model that infers latent disease subtypes and subtype-specific sequences of biomarker abnormalities by leveraging heterogeneity within cross-sectional data.^22^ By jointly modeling phenotypic diversity and ordering biomarker severity, SuStaIn enables individualized staging without requiring a priori “hypothetic” assumptions about disease subtypes. This approach has been successfully applied to neurodegenerative disorders such as Alzheimer’s disease and frontotemporal dementia using MRI-derived measures,^22,23^ synucleinopathies using autopsy data^7^ and Parkinson’s disease using multimodal imaging features.^24^

Despite these advances, important gaps remain. Progression patterns inferred from cross-sectional imaging data require longitudinal validation to determine whether inferred subtypes are stable or dynamic and whether stage advancement reflects true disease trajectories over time.^25^ To date, no study has examined whether longitudinal subtype shifts or stage velocity (e.g., ΔStage per year) derived from SuStaIn are associated with clinically meaningful neurodegeneration, such as phenoconversion in iRBD. Moreover, although pathological propagation has been characterized in postmortem studies, these approaches cannot be applied to clinical practice or repeated monitoring.^7,26^

Resting-state EEG is particularly suited for prodromal disease monitoring as it is non-invasive, cost-effective, and amenable to repeated assessment. Quantitative EEG abnormalities—particularly spectral slowing—have been associated with cognitive decline and increased risk of phenoconversion in iRBD, suggesting that EEG captures early functional alterations relevant to disease progression.

In the present study, we applied SuStaIn modeling to resting-state EEG in a well-characterized iRBD cohort with extended follow-up. By integrating cross-sectional subtype inference with longitudinal staging, we aimed to (i) identify data-driven neurophysiological subtypes and their progression patterns, (ii) assess subtype-specific clinical implications, (iii) evaluate subtype stability and stage advancement over time, and (iv) test their predictive utility for phenoconversion. This new approach establishes longitudinal EEG–based SuStaIn modeling as a scalable framework for resolving prodromal heterogeneity and dynamically predicting neurodegenerative progression, with potential implications for identifying individuals most likely to benefit from early disease-modifying interventions.

## Results

### Participant characteristics

Table 1 summarizes the demographic and clinical characteristics of the three groups. Based on the inclusion and exclusion criteria, a total of 285 participants were included in the analysis, comprising healthy controls (HC; *n* = 51) and individuals with iRBD (*n* = 234). Among the iRBD patients, 195 remained non-converted throughout the follow-up period (iRBD-NC; follow-up duration: 5.09 ± 2.53 years), whereas 39 subsequently developed an overt neurodegenerative disorder such as Parkinson’s disease, multiple system atrophy, or dementia with Lewy bodies and were classified as converters (iRBD-CV; follow-up duration: 6.23 ± 2.59 years). The follow-up duration did not significantly differ between the iRBD-NC and iRBD-CV groups (p = 0.064). The iRBD-CV group was significantly older (70.9 ± 7.5 years) than HC (66.2 ± 6.4 years, *p* = 0.004). Education level showed a decreasing trend from HC > iRBD-NC > iRBD-CV (*p* = 0.001), although the difference between HC and iRBD-NC was not statistically significant. Motor symptoms, assessed using the Movement Disorder Society–sponsored revision of the Unified Parkinson’s Disease Rating Scale Part III (MDS-UPDRS-III), were more severe in the iRBD-CV group than in the iRBD-NC group (*p* < 0.001).

Neuropsychological measures demonstrated a progressive decline across groups, with Montreal Cognitive Assessment (MoCA) scores dropping from 27.3 ± 1.5 in HC to 23.9 ± 3.5 in iRBD-CV (p < 0.001). Subjective sleep and mood parameters, including Pittsburgh Sleep Quality Index (PSQI), Epworth Sleepiness Scale (ESS), and Geriatric Depression Scale (GDS), were significantly worse in the iRBD groups compared with controls (all *p* < 0.05). RBD symptom severity, assessed using the REM Sleep Behavior Disorder Questionnaire–Korean version (RBDQ-KR), and autonomic dysfunction, evaluated using the Scales for Outcomes in Parkinson’s Disease–Autonomic (SCOPA-AUT), were both significantly higher in the iRBD groups than in controls (*p* < 0.001). No significant difference was observed in sex distribution across groups.

Between the two iRBD groups, RBD duration at baseline did not differ (iRBD-NC: 5.87 ± 6.17 years; iRBD-CV: 5.90 ± 5.31 years; p = 0.976). Age at RBD onset was also similar between groups (iRBD-NC: 61.99 ± 7.47 years; iRBD-CV: 64.92 ± 9.72 years; p = 0.057).

In the subset of participants with follow-up EEG data, the EEG follow-up duration was also comparable between the two iRBD groups (iRBD-NC: 3.84 ± 2.56 years, n = 63; iRBD-CV: 3.79 ± 2.93 years, n = 21; p = 0.944).

### Quantitative comparison of EEG patterns among healthy, iRBD-NC, and iRBD-CV groups

**Figure 1:**
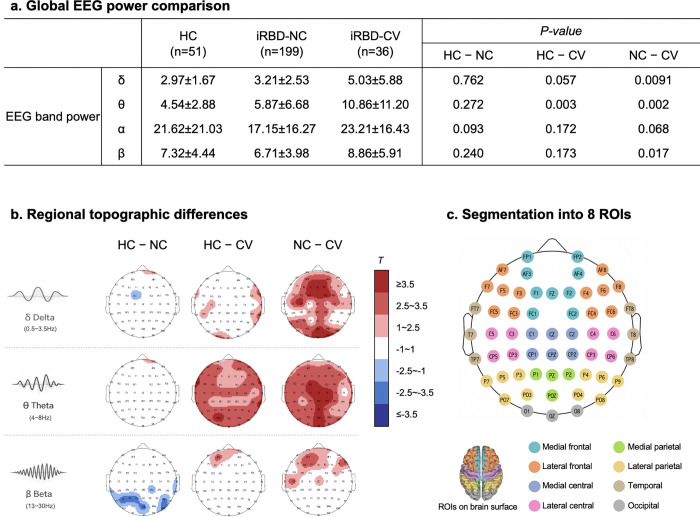
Group-level EEG spectral differences and region-of-interest definition. **a** Global EEG power differences averaged across all channels. The iRBD-CV group exhibited higher theta power than both iRBD-NC and HC, as well as elevated delta and beta power compared with iRBD-NC. **b** Channel-wise topographical maps showing significant group differences in delta, theta, and beta bands. Delta increases were most prominent over medial frontal and occipital sites, theta elevations were strongest along the medial central and parietal midline, and beta power showed an anterior–posterior dissociation. Regions showing locally reversed effects (blue) were sign-inverted to maintain consistent directionality for SuStaIn modeling. **c** Eight cortical regions of interest (ROIs) were defined—medial and lateral frontal, medial and lateral central, medial and lateral parietal, occipital, and temporal—capturing the major frequency-specific alteration patterns for subsequent analysis. For visualization purposes, ROIs were projected onto the cortical surface in Montreal Neurological Institute (MNI) space using the Automated Anatomical Labeling (AAL) atlas.

The EEG power comparison revealed distinct spectral characteristics across groups, particularly in the iRBD-CV group. As shown in figure 1a, when averaged across all channels, the iRBD-CV group exhibited higher theta power compared with both iRBD-NC and HC, as well as higher delta and beta power than iRBD-NC. In general, delta and theta power showed an increasing trend across groups (HC < iRBD-NC < iRBD-CV). In contrast, beta power did not follow this pattern, but instead showed the lowest values in iRBD-NC, intermediate values in HC, and the highest values in iRBD-CV.

These spectral differences were more clearly delineated in channel-wise topographical maps (Figure 1b). Delta power was significantly higher in iRBD-CV than in iRBD-NC, mainly over the medial frontal and occipital electrodes. Theta power was most pronounced in the iRBD-CV group, particularly over the medial central, medial parietal, and occipital regions, with the higher power along the midline. In contrast, beta power displayed an anterior–posterior dissociation: iRBD-CV showed higher beta activity in frontal regions than both HC and iRBD-NC, whereas HC exhibited relatively greater beta power than iRBD-NC in parietal and occipital areas. This pattern also explains the slightly higher mean power of iRBD-NC compared with HC in Figure 1a, although the difference was not statistically significant.

As described in the Methods section (“Subtype identification and progression pattern analysis”), regional power values were sign-inverted when disease groups exhibited lower mean power than healthy controls prior to SuStaIn modeling to ensure a consistent direction of features. Based on the observed spatial and frequency-specific patterns, eight cortical regions of interest were defined (Figure 1b): medial frontal, lateral frontal, medial central, lateral central, medial parietal, lateral parietal, occipital, and temporal. These regions captured the main frequency-specific alterations and were used as physiologically meaningful inputs for subsequent subtype and trajectory modeling.

### SuStaIn subtypes: Identification of two subtypes and their demographics and clinical characteristics

We quantified regional EEG power across eight brain regions for three frequency bands (delta, theta, and beta), resulting in a total of 24 regional power features. These features were used as input to the SuStaIn model to delineate latent disease subtypes and their corresponding progression patterns.

Based on the Cross-Validation Information Criterion (CVIC) (**Figure S1a**), model fit improved substantially as the number of subtypes increased from one to two. In contrast, further increasing the number of subtypes resulted in only minor fluctuations in CVIC, with no meaningful improvement beyond two subtypes. Consistent with this pattern, test set log-likelihood increased markedly from one to two subtypes but showed only marginal and largely comparable values across models with two to five subtypes (**Figure S1b**). Therefore, the two-subtype solution was selected for subsequent analyses, as it provided the most parsimonious balance between model complexity and discrimination power.

In addition to the two main SuStaIn-derived subtypes, we also identified a third group, namely, Subtype 0 group comprising individuals who did not advance from stage 0 due to the absence of distinguishable spectral features (all features: z<1.5). As a result, 234 iRBD patients were classified into Subtype 0 (n = 119), Subtype 1 (n = 51), and Subtype 2 (n = 64). Demographic and clinical characteristics of the three subtypes are summarized in Table 2.

Subtype 1 showed a significantly higher proportion of males than Subtype 0 and Subtype 2 (Subtype 1: 78.4% vs. Subtype 0: 62.2%, p=0.039, Subtype 2: 46.2%, p=0.0006). Subtype 1 also had a significantly higher mean education level (vs. Subtype 0: p=0.033; vs. Subtype 2: p=0.024) and mean MoCA score than Subtype 0 and Subtype 2 (vs. Subtype 0: p=0.034; vs. Subtype 2: p=0.003). On the other hand, Subtype 2 exhibited greater Epworth Sleepiness Scale (ESS) than Subtype 1 (p=0.03).

No other demographic, cognitive, sleep-related, or mood-related measures differed significantly across the three subtypes.

### SuStaIn subtypes: Neurophysiological progression

**Figure 2:**
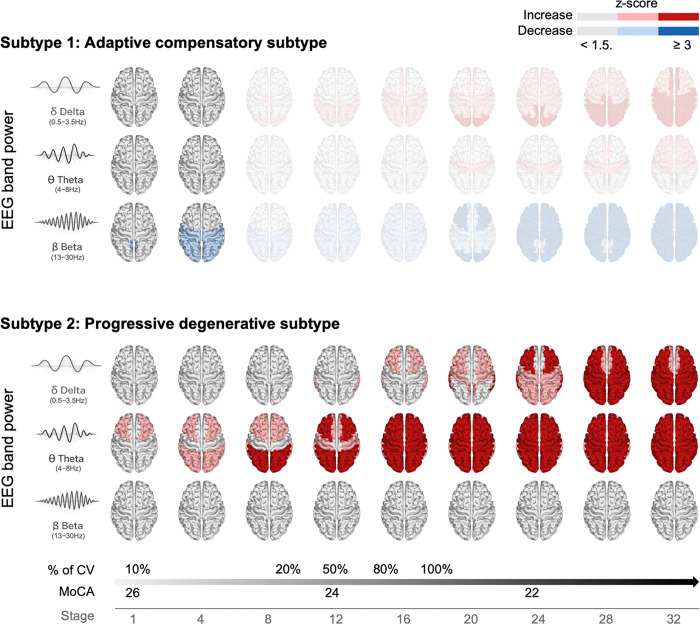
Stage-dependent neurophysiological progression across SuStaIn-defined iRBD subtypes. Stage-wise alterations reflect the SuStaIn model–estimated progression of HC-normalized z-scores across stages. Light red and blue indicate z-scores between 1.5 and 3, whereas saturated colors indicate z-scores ≥ 3. Beta power values that showed reductions in the posterior area relative to HC were sign-inverted prior to model fitting to ensure a consistent unidirectional progression of abnormality. Accordingly, blue regions reflect abnormalities characterized by reduced power relative to HC, whereas red regions represent abnormalities associated with increased power. Subtype 1 exhibited early, spatially restricted posterior beta abnormalities localized primarily to occipital regions, which emerged at initial stages (stages 1–4) and did not demonstrate further spatial expansion or additional frequency involvement across later stages. In contrast, Subtype 2 showed a progressive and spatially propagating pattern, beginning with theta abnormalities in lateral frontal regions, followed by delta involvement in lateral frontal and temporal cortices, and ultimately extending to widespread cortical regions with advancing stage. No beta abnormalities were observed across stages in Subtype 2. Semi-transparent stages represent model-inferred extrapolated stages without assigned participants and should be interpreted cautiously. The proportion of phenoconverters increased with stage advancement (logistic regression: OR/5-stage = 2.14, p = 0.009), and cognitive performance (MoCA) declined along the stage trajectory in Subtype 2 (logistic regression: OR/5-stage = 2.14, p = 0.009).

The spectral changes of the two subtypes with stage advancement are shown in Figure 2. With stage advancement, the two subtypes exhibited markedly different spectral trajectories. Subtype 1 showed abnormal beta activity limited to posterior regions, primarily in the occipital cortex, which appeared at early stages (stage 1–4) and did not further expand or evolve with other power activities across stages. In contrast, Subtype 2 demonstrated a progressive pattern characterized by early abnormal theta activity emerging from the lateral frontal regions, followed by the appearance of delta abnormalities in the lateral temporal and frontal areas. These slow-wave changes gradually extended to widespread cortical regions as the stage increased. No beta abnormalties were observed across stages in Subtype 2.

Patients with iRBD-CV were present exclusively in Subtype 2, and their proportion increased progressively with stage advancement (p = 0.009). In terms of clinical outcomes, MoCA scores significantly decreased along with stage progression in Subtype 2 (p = 0.015), while no other clinical or neurophysiological measures showed significant associations with stage advancement (see **Figure S2**).

### Analysis of prospective phenoconversion among SuStaIn subtypes

**Figure 3:**
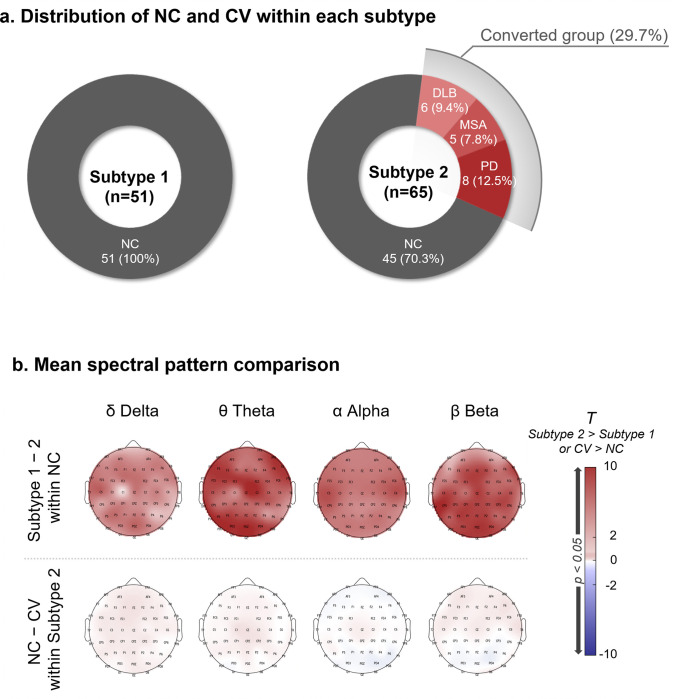
Characteristics of two EEG-based iRBD subtypes in terms of distribution of phenoconverters and spectral profiles. **a** Subtype 1 (n = 51) comprised exclusively iRBD-NC participants. In contrast, Subtype 2 (n = 64) included both iRBD-NC (45/64, 70.3%) and iRBD-CV (19/64, 29.7%) individuals who subsequently developed Parkinson’s disease (PD, n = 8), multiple system atrophy (MSA, n = 5), or dementia with Lewy bodies (DLB, n = 6). Converters were observed exclusively in Subtype 2. **b**Spectral comparisons between iRBD-NC participants across subtypes demonstrated higher delta, theta, alpha, and beta power in Subtype 2 across most cortical regions, indicating subtype-specific neurophysiological differences independent of conversion status. Within Subtype 2, no significant spectral differences were observed between iRBD-NC and iRBD-CV participants, suggesting that EEG divergence is primarily driven by between-subtype distinctions rather than conversion status within Subtype 2.

As shown in Figure 3a, Subtype 1 comprised exclusively iRBD non-converters (iRBD-NC; *n* = 51), whereas Subtype 2 included 45 iRBD-NC (70.3%) and 19 iRBD converters (iRBD-CV; 29.7%) who subsequently developed PD (*n* = 8), MSA (*n* = 5), or DLB (*n* = 6). The prevalence of phenoconversion was significantly higher in Subtype 2 than in Subtype 1 (Fisher’s exact test, *p* = 4.35 10^−6^), indicating that Subtype 2 represents a more progressive prodromal phenotype.

To determine whether non-converted individuals within Subtype 2 nevertheless exhibited greater neurophysiological progression than those in Subtype 1, we compared mean regional spectral power between iRBD-NC patients from the two subtypes. Subtype 2 non-converters demonstrated significantly higher delta, theta, alpha, and beta power across nearly all cortical regions (Figure 3b), suggesting that the two subtypes reflect fundamentally distinct electrophysiological profiles independent of conversion status. However, when comparing clinical characteristics of non-converters between Subtype 1 and 2, there were no significant differences (**Table S1**).

We further examined whether phenoconversion status was associated with additional EEG differences within Subtype 2 by comparing iRBD-NC and iRBD-CV patients classified in this subtype. No significant spectral differences were observed between these groups, indicating that EEG divergence is primarily driven by between-subtype distinctions rather than conversion status within Subtype 2.

### Longitudinal neurophysiological progression

To evaluate the longitudinal validity of the SuStaIn model, we assessed (1) whether iRBD patients remained in the same subtype at follow-up and (2) whether patients display stage advancement or retreat relative to baseline. This analysis included participants with a single follow-up assessment (Subtype 0: *n* = 43, Subtype 1: *n* = 18, Subtype 2: *n* = 23). Baseline–follow-up EEG intervals did not differ significantly across subtypes (Table 2).

**Figure 4:**
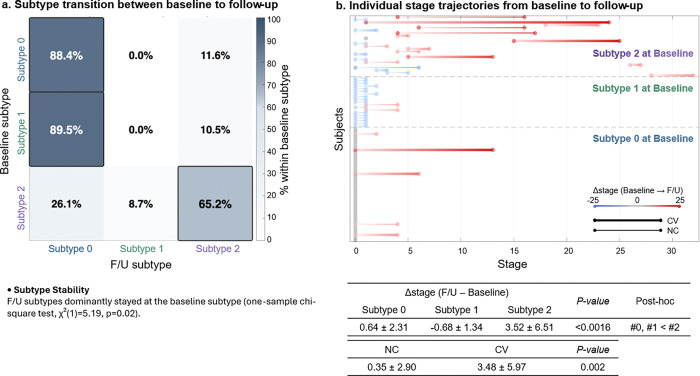
Longitudinal validation of SuStaIn subtype stability and stage progression. **a** Baseline-to-follow-up subtype transition matrix. The majority of participants classified as Subtype 0 (88.4%) and Subtype 2 (65.2%) remained in their baseline subtype at follow-up. In contrast, Subtype 1 predominantly transitioned to Subtype 0 (89.5%), with 10.5% shifting to Subtype 2. Among Subtype 2 participants, 26.1% transitioned to Subtype 0 and 8.7% to Subtype 1. Overall, retention of baseline subtype occurred more frequently than expected by chance (one-sample χ^2^(1) = 5.19, p = 0.02). **b** Individual longitudinal SuStaIn stage trajectories from baseline to follow-up. Red and blue lines indicate stage advancement and retreat, respectively. The largest stage advancement was observed in Subtype 2 (mean Δstage = 3.52 ± 6.51). iRBD-CV group (thick lines) demonstrated significantly greater stage advancement than iRBD-NC (thin line, 3.48 ± 5.97 vs. 0.35 ± 2.90, p = 0.002).

As shown in Figure 4a, among participants initially classified as Subtype 0, 88.4% remained in the same subtype, while 11.6% transitioned to Subtype 2. In Subtype 2, 65.2% retained their classification, whereas 26.1% transitioned to Subtype 0 and 8.7% to Subtype 1. In contrast, Subtype 1 demonstrated a distinct transition pattern: 89.5% shifted to Subtype 0 and 10.5% to Subtype 2 at follow-up. Across all participants, retention of baseline subtype at follow-up occurred significantly more frequently than expected by chance (one-sample chi-square, *χ*^*2*^*(1)* = 5.19, *p* = 0.02), supporting the longitudinal robustness of subtype classification.

Longitudinal changes in SuStaIn stage are illustrated in Figure 4b, with red and blue lines indicating stage advancement and retreat, respectively. Across the cohort, mean stage increased by 1.13 ± 4.09 (*p* = 0.0066), indicating overall progression. Subtype 0 participants largely remained at stage 0 (88.4%), except for a subset (11.6%) who advanced in stage and transitioned to Subtype 2, primarily among converters. Subtype 1 participants predominantly regressed toward stage 0 in parallel with transition to Subtype 0 (89.5%). In contrast, Subtype 2 exhibited significant stage advancement, with a mean increase of 3.52 ± 6.51, consistent with a progressive trajectory.

Across all participants, converters demonstrated significantly greater stage advancement than non-converters (3.48 ± 5.97 vs. 0.35 ± 2.90, *p* = 0.002), indicating that SuStaIn stage progression aligns with clinical phenoconversion.

### Prediction of 7.5-year cumulative phenoconversion by SuStaIn EEG subtypes and stage progression

**Figure 5:**
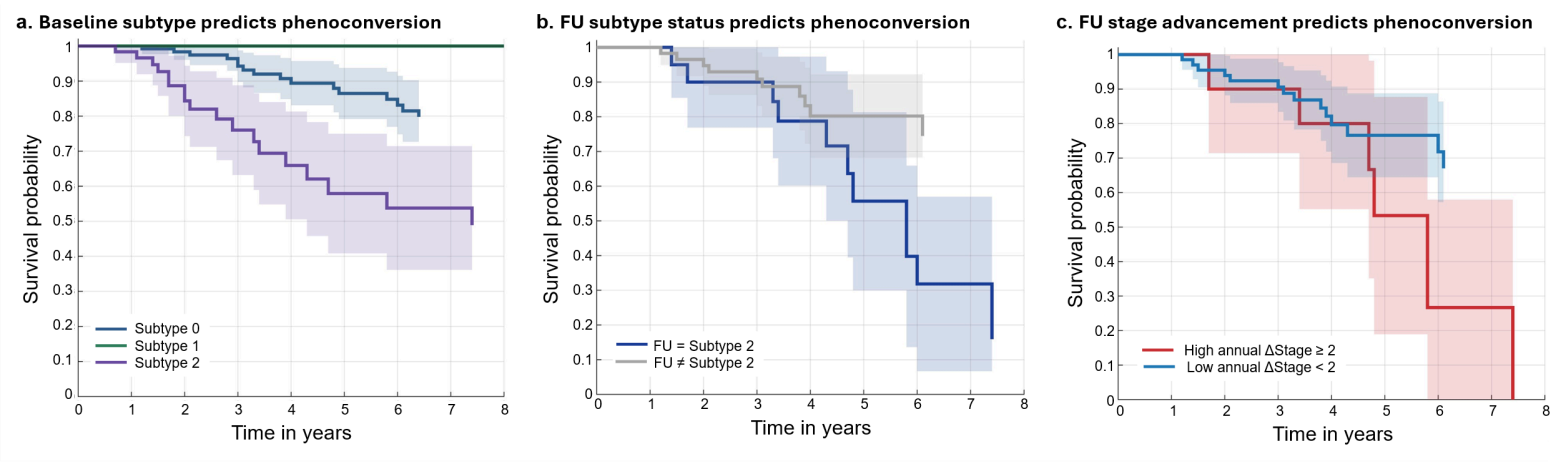
Prediction of 7.5-year phenoconversion by SuStaIn subtypes and stage progression. Kaplan–Meier survival curves illustrating conversion-free survival across subtype classifications and longitudinal progression metrics. **a** Survival prediction by baseline subtype classification. Subtype 1 showed no phenoconversion events, Subtype 0 demonstrated intermediate risk, and Subtype 2 exhibited the lowest survival probability. **b** Follow-up subtype status. Individuals who remained in or transitioned to Subtype 2 at follow-up showed significantly reduced conversion-free survival compared with those classified as Subtype 0 or 1. **c**Longitudinal stage advancement. Participants with accelerated annual stage progression (ΔStage ≥ 2 per year) exhibited markedly reduced survival compared with those with slower progression (ΔStage < 2). By approximately 7.5 years, conversion-free survival approached zero in the faster progression group. Shaded areas represent 95% confidence intervals.

We evaluated whether EEG-based SuStaIn subtype classification and longitudinal stage progression predicted risk of phenoconversion over up to 7.5-year follow-up period using Kaplan–Meier survival analysis (Figure 5).

Baseline subtype classification (Figure 5a): Conversion-free survival differed markedly across baseline subtypes (global log-rank *p* = 4.86 × 10^−7^). Individuals classified as Subtype 1 demonstrated 100% survival throughout follow-up, with no phenoconversion events observed. Subtype 0 showed intermediate risk, with survival probability declining to approximately 80% by 6 years. In contrast, Subtype 2 exhibited substantially reduced survival probability, decreasing to approximately 50% at 7.5 years. Pairwise comparisons confirmed significant differences between Subtype 0 and Subtype 1 (*p* = 0.026), Subtype 0 and Subtype 2 (*p* = 4.75 × 10^−5^), and Subtype 1 and Subtype 2 (*p* = 1.68 × 10^−5^).

Follow-up subtype status (Figure 5b): Stratification based on subtype status at follow-up (mean interval = 3.83 years) further amplified risk discrimination. Individuals who remained in or transitioned to Subtype 2 exhibited sharply reduced conversion-free survival, declining to approximately 15% by 7.5 years. In contrast, individuals classified as Subtype 0 or 1 at follow-up maintained substantially higher survival probabilities (75% by 6.1 years; *p*=0.016).

Longitudinal stage advancement (Figure 5c): We further examined whether the rate of SuStaIn stage progression predicted phenoconversion. Participants with high annual stage advancement (ΔStage ≥ 2 per year) demonstrated dramatically reduced conversion-free survival rate (0% survival rate by 7.5 years) compared with those with slower progression (67% survival rate by 6.1 years; *p* = 0.032).

## Discussion

In our study with longitudinal EEG follow-up, we applied the SuStaIn framework to resting-state EEG in iRBD and identified biologically meaningful prodromal biotypes with distinct progression trajectories. This modeling identified two biologically distinct prodromal biotypes: an adaptive–compensatory subtype (Subtype 1) and a progressive–degenerative subtype (Subtype 2), in addition to Subtype 0, which exhibited no detectable EEG abnormalities. The adaptive–compensatory subtype (Subtype 1) was characterized by a higher proportion of males, greater educational attainment, and better cognitive performance, consistent with demographic differences in educational opportunity in older male populations and with the well-established protective effects of education and cognitive reserve on disease progression and cognition. Longitudinal validation demonstrated that SuStaIn staging distinguishes stable trajectories (Subtypes 0 and 1) from progressive trajectories (Subtype 2), with stage advancement strongly linked to phenoconversion. These findings support EEG-based SuStaIn modeling as a scalable framework for stratifying prodromal heterogeneity and neurodegenerative risk in iRBD, thereby providing insights into individually varying time window for therapeutic intervention.

This is the first study to extend SuStaIn modeling to EEG-derived functional biomarkers and to directly validate inferred subtypes and stages using longitudinal EEG data and long-term phenoconversion outcomes. Whereas prior SuStaIn applications have focused primarily on MRI-derived structural measures^26^ or autopsy data,^7,26^ EEG provides a non-invasive and longitudinally scalable modality that reflects neuronal and synaptic activity and may capture functional alterations preceding structural degeneration.^22^ Importantly, EEG-based SuStaIn identified distinct prodromal biotypes even among patients who were clinically indistinguishable at baseline, highlighting its potential as an early functional marker of disease trajectory. These findings were not revealed in prior work that has primarily focused on the hypothetical brain-first and body-first phenotypic model.^26,27^

The first key finding was the identification of two EEG-based prodromal biotypes with distinct spatial and temporal patterns. Subtype 1 was characterized by an isolated reduction in posterior beta power, predominantly localized to occipital regions without anterior propagation. This isolated beta reductions in prodromal RBD phenotypes without accompanying delta/theta increases align with recent work by Terranova et al.(2024)^28^. However, other prior studies have had limited interpretation of iRBD heterogeneity: Posterior beta reductions were typically observed alongside anterior slowing, obscuring their potential role as an early and independent subtype-defining feature.^29^ More recent resting-state EEG studies have similarly reported increased rhythmic theta activity accompanied by reductions in fast oscillations within occipital regions. A large baseline analysis of EEG spectral components and functional connectivity in iRBD demonstrated reduced occipital alpha power together with enhanced theta activity, interpreted as disruption of posterior networks implicated in default mode network (DMN) regulation.^30^ Metabolic imaging studies similarily showed simultaneous increased and decreased metabolic activities. While the increased metabolism was found in the pons, thalamus, precentral gyrus, supplementary motor area, medial frontal gyrus, and cerebellum, the decreased metabolic activity occurred in occipital regions.^31^

In contrast, Subtype 2 showed progressive cortical slowing initially involving frontal regions and eventually expanded to posterior cortical areas and was the only group to exhibit cognitive deficits. This spatiotemporal progression in iRBD was more pronounced in patients with phenoconversion. Frontal-predominant delta/theta hyperactivity, followed by posterior involvement, is a well-established electrophysiological hallmark of emerging synucleinopathy. Prior studies have shown that RBD patients with global EEG slowing are at increased risk of cognitive decline and dementia,^32,33^ which was in line with our observation in Subtype 2 displaying significantly lower MoCA scores and encompassed all converters.

This pattern aligns closely with the known cognitive and neuroanatomical signatures of Lewy body disorders. Compared with Alzheimer’s disease, DLB and PD dementia are characterized by disproportionate impairments in attention, executive, and visuospatial domains^34^. Neuroimaging studies further demonstrate preferential involvement of frontal, parieto-temporal, and occipital cortices with relative hippocampal sparing.^35,36^ Notably, a recent MRI-based SuStaIn analysis identified a “cortical-first” iRBD subtype marked by early frontal atrophy with gradual posterior spread, which was associated with faster cognitive decline and a higher risk of conversion to DLB.^37^ Our EEG-derived Subtype 2 closely parallels this cortical-first trajectory, exhibiting early frontal delta/theta hyperactivity, more severe cognitive impairment, and rapid progression to overt synucleinopathy. This concordance across modalities supports that Subtype 2 reflects a malignant prodromal pathway characterized by frontal-to-posterior network degeneration, conferring heightened vulnerability to DLB.

Longitudinally, subtype assignments were largely stable. Subtype 0 remained unchanged in most individuals. Subtype 2 demonstrated the greatest persistence and stage advancement, whereas Subtype 1 predominantly transitioned back to Subtype 0, reflecting normalization of posterior beta abnormalities. This pattern aligns with prior prospective cohort studies showing that iRBD patients who phenoconvert accumulate motor and cognitive deficits more rapidly than those who remain stable, with steeper declines in disease-free survival and higher hazard ratios in converters.^38^

Furthermore, nearly 90% of patients of Subtype 1 reverted to Subtype 0 at follow-up. This reversible pattern closely parallels longitudinal FDG-PET studies in iRBD showing attenuation of occipital hypometabolism and relative increases in frontal metabolism over time, consistent with compensatory fronto–occipital network dynamics ).^39–41^ The fronto-occipital circuit dysfunction hypothesis suggests that early occipital changes may occur in response to subcortical stress, but compensatory mechanisms (likely in response to frontal hyperactivation) preserve function as disease progresses. In other words, Subtype 1 may embody a transient compensatory state: despite early posterior stress (α-synuclein burden in visual cortex or cholinergic afferents), patients maintain cognition and clinical stability.

Importantly, the prognostic value of EEG-based SuStaIn modeling was substantial. Baseline subtype classification alone stratified long-term conversion risk, with Subtype 2 demonstrating markedly reduced 7.5-year conversion-free survival (50%) compared with Subtype 0 (80%) and complete survival in Subtype 1. Risk discrimination increased further when follow-up subtype status was considered (Subtype 2 survival: approximately 15% by 7.5 years vs. Subtype 0–1: 75%). Most strikingly, Individuals demonstrating accelerated annual stage advancement (ΔStage ≥ 2 per year) exhibited no survival by 7.5 years, compared with approximately 70% survival among those with slower progression. This degree of separation suggests that EEG-based SuStaIn captures not only iRBD subtypes but also the *dynamic* subtype switching and neurophysiological deterioration, identifying individuals at exceptionally high risk of conversion and providing a clinically actionable biomarker for trial enrichment and individualized risk monitoring.

Demographic differences across EEG-defined subtypes should be interpreted within the broader biological, historical, and sociocultural context of older clinical cohorts. Although Subtype 1 included a higher proportion of males and individuals with greater educational attainment, mean age did not differ significantly across subtypes, indicating that age does not account for subtype-specific trajectories. In older generations, male sex is associated with greater access to formal education,^42^ a relationship that was also evident in our cohort (fisher’s exact test: p=0.029). Accordingly, the demographic profile of Subtype 1 is more plausibly driven by educational attainment and cognitive reserve, rather than by biological sex itself. Higher education is a well-established proxy for cognitive and neural reserve in the context of Alzheimer’s and related dementia.^43,44^ Thus, the relatively preserved cognition and phenoconversion as well as frequent normalization of EEG abnormalities in Subtype 1 are likely explained by reserve-mediated compensation. Subtype 1 also displayed a marginally earlier RBD onset compared with Subtype 2 (p = 0.05), raising the possibility that earlier disease onset combined with higher educational attainment confers a cumulative neuroprotective effect.^45,46^ In contrast, a higher proportion of females and faster neurodegenerative progression in Subype 2 possibly relate to diminish of estrogen-related neuroprotective effects after menopause.^47–49^ However, this interpretation remains speculative and secondary to reserve-related explanations since neurodegenerative biomarkers in iRBD have generally shown limited or inconsistent sex effects.^50^

This study has several limitations. First, it was conducted at a single center in a single cohort, which may limit generalizability. Although participants were prospectively recruited and followed using standardized clinical and EEG protocols over an extended period, replication in independent, multicenter cohorts is necessary to confirm the robustness and external validity of the identified EEG-based subtypes and trajectories. Second, EEG primarily reflects cortical neurophysiological activity and provides limited direct information about subcortical structures—such as the brainstem, basal ganglia, and thalamus—that are central to iRBD and synucleinopathy pathophysiology.^51^ Multimodal studies integrating EEG with structural MRI, dopaminergic imaging, or PET will be important to better characterize disease propagation. Third, limitations inherent to the SuStaIn framework should be considered. SuStaIn infers progression from cross-sectional variability and assumes monotonic biomarker change. We addressed these issues by harmonizing the directionality of EEG features and validating the model using longitudinal follow-up and phenoconversion outcomes. Fourth, although phenoconversion represents a clinically meaningful endpoint, the number of pheoconverters was relatively small, limiting subtype-specific analyses across individual α-synucleinopathies.

In conclusion, this study demonstrates that resting-state EEG–based SuStaIn modeling delineates biologically meaningful prodromal subtypes and stage trajectories in isolated REM sleep behavior disorder. By integrating functional EEG biomarkers with a data-driven disease progression framework and validating inferred stages using longitudinal phenoconversion outcomes, we show that early neurophysiological alterations capture clinically relevant heterogeneity in iRBD. Distinct trajectories were identified, including a subtype characterized by isolated and reversible posterior beta alterations suggestive of compensatory network responses, and a progressive subtype marked by frontal slow-wave propagation and cognitive decline. Importantly, EEG-based staging differentiated clinically stable individuals from those at increased risk of neurodegenerative progression, with converters exhibiting accelerated stage advancement. These findings establish EEG-informed SuStaIn staging as a practical and scalable approach for early risk stratification and disease monitoring in prodromal synucleinopathy, with potential implications for patient selection and timing of disease-modifying interventions.

## Methods

### Participants

Participants were recruited from Seoul National University Hospital between October 2017 and January 2025 as part of a prospective single-center cohort study.^52^ In this cohort, iRBD was diagnosed according to the International Classification of Sleep Disorders—Third Edition (ICSD-3) criteria using overnight video-polysomnography (vPSG).^1^ Two board-certified neurologists specialized in sleep medicine (K.Y. Jung) and movement disorders (H.J. Kim) conducted baseline evaluations to screen for dementia, cerebellar ataxia, parkinsonism, and other neurodegenerative disorders. Individuals with an established neurodegenerative disease, neurological or severe medical illness, were excluded.

Phenoconversion assessments were performed every 6 months following established diagnostic criteria.^53–55^ Patients who developed PD, DLB, or MSA during follow-up were classified as iRBD-CV; those who remained free of phenoconversion were categorized as iRBD-NC.

HC with MoCA-K scores > 26 were also enrolled. This study was approved by the Institutional Review Board of Seoul National University Hospital (IRB Nos. 1708–169-883 and 2503–118-1623), and written informed consent was obtained from all participants.

### Outcome variables

Motor and cognitive functions were assessed using MDS-UPDRS-III, the Korean version of the Mini-Mental Status Examination (MMSE), and the Korean version of the MoCA.^56–58^

RBD symptom severity was measured using RBDQ-KR.^59^ Olfactory function was assessed with the Korean Version of Sniffing Sticks (KVSS).^60^ Autonomic symptoms were evaluated using the SCOPA-AUT.^61^ Subjective sleep quality and daytime sleepiness were evaluated with the PSQI and ESS, respectively.^62,63^ Depressive symptoms were assessed using the GDS.^64^

During follow-up, annual evaluations included cognitive function (MoCA), motor impairment (MDS-UPDRS-III), autonomic dysfunction (SCOPA-AUT), subjective sleep quality and daytime sleepiness (PSQI, ESS), RBD symptom severity (RBDQ-KR), depressive symptoms, and olfactory function (KVSS).

### EEG data acquisition and preprocessing

In a cohort, participants underwent baseline EEG assessments, and patients with iRBD had biannual EEG follow-up assessments. For Resting-state EEGs were recorded using a 58-channel EEG cap arranged according to the international 10–10 electrode system. The reference electrode was placed on one ear, and the ground electrode was located at AFz. Electrode impedances were maintained below 10 kΩ throughout the recording. Each participant underwent a 5-minute resting-state EEG session while awake in a quiet, dimly lit room. Participants were instructed to remain relaxed and alternate between eyes open and eyes closed every 30 seconds. Only the eyes-closed segments were extracted and analyzed to minimize visual alpha modulation. After preprocessing and artifact rejection, approximately 101 seconds of clean EEG data per participant were retained for the final analysis. EEG signals were digitized at a sampling rate of 400 Hz, and a 0.5 Hz high-pass filter and 60 Hz notch filter were applied to remove slow drifts and power-line noise.

All recordings were visually inspected by trained technicians blinded to group information, and segments contaminated by movement, muscle, or ocular artifacts were removed. Independent component analysis (ICA) was performed using EEGLAB v2019.1 (MATLAB R2020b, The MathWorks, Natick, MA, USA), and components representing ocular activity were automatically identified and rejected using the ICLabel plugin with a 90% probability threshold. Only participants with at least 80% artifact-free data were included in the final analysis. For spectral quantification, absolute band power was computed for the delta (0.5–3.5 Hz), theta (4–8 Hz), alpha (8–13 Hz), and beta (13–30 Hz) frequency bands. Band power was estimated by integrating the power spectral density (PSD) over each frequency range, where the PSD was obtained using Welch’s averaged periodogram method.

### Subtype identification and progression pattern analysis

To characterize heterogeneity in EEG alterations and their potential disease progression, we employed the SuStaIn model, which captures distinct progression trajectories from cross-sectional data by simultaneously estimating latent subtypes and their sequential biomarker changes.

Observer-guided EEG features were first defined based on Figure 1a–c. Frequency bands that showed the most significant group differences in Figure 1a, including delta (0.5–3.5Hz), theta (4–8 Hz), and beta (13–30 Hz), were selected to reduce feature dimensionality. The spatial definition of regions of interest (ROIs) was guided by the regional patterns observed in Figure 1b, where lateral and medial areas exhibited distinct alteration profiles.^40^ Based on these spatial characteristics and their clinical interpretability, eight ROIs were defined as frontal medial, frontal lateral, central, parietal medial, parietal lateral, occipital, temporal lateral, and temporal basal regions, as summarized in Figure 1c. Band powers were averaged within each ROI, yielding a total of 24 features (three frequency bands × eight ROIs) for each participant.

Because SuStaIn requires each feature to vary monotonically along the disease progression axis, regional power values that exhibited locally opposite changes relative to controls were corrected prior to model fitting. As shown in Figure 1b, while the iRBD groups showed overall higher values of spectral power, several regions in the iRBD-NC group exhibited relatively lower power compared with controls, indicating that the abnormality pattern was not strictly unidirectional. For these regions, the sign of the power values was inverted prior to SuStaIn modeling. Thus, all features consistently represented deviation in the same direction of abnormality, thereby ensuring coherent progression modeling.

After adjusting for age and sex based on HCs, the corrected 24 feature values were standardized as z-scores and used as inputs to the SuStaIn model. The model estimated distinct EEG-based subtypes and their corresponding progression trajectories. The optimal number of subtypes was determined using the CVIC and out-of-sample log-likelihood through five-fold cross-validation,^65^ in which 80% of the data were used for training and 20% for testing in each iteration. Finally, subtype-specific progression trajectories were characterized by projecting all participants onto the inferred SuStaIn stage axis, and the distributions of iRBD-NC and iRBD-CV cases across subtypes and stages were analyzed to assess how EEG-derived progression patterns corresponded to clinical conversion and longitudinal outcomes.

### Statistical analysis

All statistical analyses were performed using MATLAB R2020a (The MathWorks, Natick, MA, USA). Group-level comparisons (HC vs. iRBD-NC vs. iRBD-CV) of demographic, clinical, and EEG variables were conducted using analysis of covariance (ANCOVA) for continuous measures, with age and sex included as covariates, and chi-square tests for categorical measures. When significant main effects were observed, Bonferroni-corrected post hoc tests were performed.

For the SuStaIn-derived subtype analyses, subtype membership distributions were compared using chi-square tests. Continuous clinical and EEG variables were compared across subtypes using one-way ANOVA or independent t-tests depending on the number of groups being contrasted, while categorical variables were evaluated using chi-square tests.

Longitudinal validity was examined by evaluating subtype stability across follow-up using cross-tabulation and chi-square analysis, and by assessing changes in SuStaIn stage using paired t-tests comparing baseline and follow-up values. Time-to-event analyses were conducted using Kaplan–Meier survival curves to estimate conversion-free survival, and differences between survival curves were assessed using log-rank tests. To control for multiple comparisons, p-values were adjusted using the false discovery rate (FDR) procedure.

## Supplementary Material

Supplementary Files

This is a list of supplementary files associated with this preprint. Click to download.

• SupplementaryFN.docx

## Figures and Tables

**Table 1.** Demographic and clinical characteristics of study participants. Summary of demographic, clinical, and neuropsychological variables among HC, iRBD-NC, and iRBD-CV groups. iRBD-CV participants were significantly older and less educated than other groups and showed higher MDS-UPDRS-III scores. MoCA scores progressively decreased from HC to iRBD-CV, whereas sleep and mood parameters (PSQI, ESS, GDS) were worse in both iRBD groups relative to controls. RBDQ-KR and SCOPA-AUT were markedly elevated in iRBD compared with HC.

	HC^a^ (n = 51)	iRBD-NC^b^ (n = 195)	iRBD-CV to PD/MSA/DLB^c^ (n = 39)	*P-value*	Post-Hoc
Age, yr	66.22 ± 6.37	67.85 ± 6.60	70.90 ± 7.51	0.011	a<c
Male, %	68.6	63.1	52.5	0.433	
Education, yr	13.69 ± 2.66	12.87 ± 3.90	10.36 ± 4.26	0.001	c<a, b
MDS-UPDRS-III	-	1.01 ± 2.19	3.35 ± 5.71	<0.001	b<c
KVSS	0.63 ± 0.49	1.70 ± 3.41	1.85 ± 3.46	0.329	
MMSE	28.88 ± 0.96	27.93 ± 1.87	27.33 ± 1.98	0.059	
MoCA	27.33 ± 1.47	26.07 ± 2.80	23.85 ± 3.54	<0.001	c<b<a
RBDQ-KR	4.86 ± 3.37	40.31 ± 18.59	42.86 ± 21.61	<0.001	a<b,c
ESS	4.12 ± 2.72	5.08 ± 3.48	6.41 ± 4.42	0.024	a<b,c
GDS	4.14 ± 4.88	9.00 ± 5.91	11.27 ± 6.40	<0.001	a<b,c
PSQI	3.35 ± 1.53	7.28 ± 4.12	6.03 ± 3.57	<0.001	a<b,c
SCOPA-AUT	5.90 ± 4.31	13.09 ± 7.75	13.47 ± 7.98	<0.001	a<b,c
RBD duration at baseline, yr	-	5.87 ± 6.17	5.90 ± 5.31	0.976	
Age at RBD onset, yr	-	61.99 ± 7.47	64.92 ± 9.72	0.057	
Cohort F/U duration, yr	-	5.09 ± 2.53	6.23 ± 2.59	0.064	
EEG F/U duration, yr	-	3.84 ± 2.56 (n=63)	3.79 ± 2.93 (n=21)	0.976	

Abbreviations: iRBD-NC, Non-Converted idiopathic Rapid eye movement sleep Behavior Disorder; iRBD-CV, Converted idiopathic Rapid eye movement sleep Behavior Disorder; ESS, Epworth Sleepiness Scale; F/U, Follow-Up; GDS, Geriatric Depression Scale; KVSS, Korean Version of the Sniffin' Sticks Test; MMSE, Mini-Mental State Examination; MoCA, Montreal Cognitive Assessment; PSQI, Pittsburgh Sleep Quality Index; RBDQ-KR, Rapid Eye Movement Sleep Behavior Disorder Questionnaire-Korean version; SCOPA-AUT, Scales for Outcomes in Parkinson's Disease-Autonomic; MDS-UPDRS-III, Movement Disorder Society-sponsored revision of the Unified Parkinson's Disease Rating Scale Part III

**Table 2.** Comparison of demographic and clinical variables across iRBD subtypes. Demographic and clinical characteristics across Subtypes 0, 1, and 2 identified by SuStaIn. Subtype 1 showed a higher proportion of males, greater education levels, and better cognitive function (MoCA) compared with Subtypes 0 and 2, whereas no significant differences were found in age or sleep-related measures.

	Subtype 0 (n=119)	Subtype 1 (n=51)	Subtype 2 (n=64)	Sutype0 > Subtype 1&2		Subtype 0 > Subtype 1		Subtype 0 > Subtype 2		Subtype 1 > Subtype 2	
				*T-value*	*P-value*	*T-value*	*P-value*	*T-value*	*P-value*	*T-value*	*P-value*
Age, yr	67.78 ± 7.07	68.00 ± 6.51	69.72 ± 6.58	−1.33	0.185	−0.20	0.846	−1.86	0.065	−1.41	0.162
Male, %	62.2	78.4	46.2		0.836		**0.039**		**0.046**		**0.0006**
Education	12.24 ± 4.13	13.57 ± 3.44	11.90 ± 4.29	−0.78	0.439	−2.16	**0.033**	0.51	0.614	2.29	**0.024**
MDS-UPDRS-III	1.44 ± 3.72	1.02 ± 2.10	1.47 ± 2.51	0.40	0.687	0.87	0.385	−0.04	0.966	−0.99	0.326
KVSS	1.92 ± 3.53	1.56 ± 3.35	1.47 ± 3.25	0.79	0.429	0.56	0.575	0.74	0.464	0.13	0.896
MMSE	27.69 ± 2.04	28.30 ± 1.61	27.64 ± 1.81	−0.93	0.352	−1.94	0.056	0.14	0.889	1.89	0.062
MoCA	25.61 ± 3.14	26.65 ± 2.64	24.98 ± 3.07	−0.30	0.767	−2.14	0.034	1.24	0.216	3.01	**0.003**
RBDQ-KR	39.42 ± 18.84	43.86 ± 19.24	40.48 ± 19.39	−0.99	0.324	−1.35	0.182	−0.34	0.732	0.91	0.363
ESS	5.09 ± 3.16	4.61 ± 3.32	6.25 ± 4.59	−0.11	0.366	0.85	0.397	−1.79	0.077	−2.19	**0.030**
GDS	9.33 ± 5.61	8.48 ± 6.64	10.18 ± 6.25	−1.19	0.913	0.79	0.434	−0.88	0.379	−1.38	0.171
PSQI	6.74 ± 3.73	7.45 ± 4.39	7.33 ± 4.34	−1.26	0.236	−0.98	0.328	−0.91	0.364	0.14	0.890
SCOPA-AUT	12.49 ± 7.52	13.36 ± 8.65	14.16 ± 7.47	−0.72	0.210	−0.62	0.540	−1.40	0.164	−0.52	0.606
RBD onset age, yr	62.18 ± 7.79	61.14 ± 8.04	64.12 ± 7.96	−0.63	0.532	0.77	0.442	−1.60	0.113	−1.978	0.050
RBD duration, yr	5.61 ± 5.39	6.92 ± 8.25	5.60 ± 5.00	−0.72	0.473	−1.04	0.303	0.01	0.995	1.00	0.321
EEG F/U duration, yr	3.98 ± 2.56 (n=43)	4.44 ± 2.38 (n=18)	3.07 ± 2.92 (n=23)	0.52	0.60	−0.68	0.50	1.25	0.22	1.66	0.11

Abbreviations: ESS, Epworth Sleepiness Scale; F/U, Follow-Up; GDS, Geriatric Depression Scale; KVSS, Korean Version of the Sniffin' Sticks Test; MMSE, Mini-Mental State Examination; MoCA, Montreal Cognitive Assessment; PSQI, Pittsburgh Sleep Quality Index; RBDQ-KR, Rapid Eye Movement Sleep Behavior Disorder Questionnaire-Korean version; SCOPA-AUT, Scales for Outcomes in Parkinson's Disease-Autonomic; MDS-UPDRS-III, Movement Disorder Society-sponsored revision of the Unified Parkinson's Disease Rating Scale Part III

## Data Availability

The data that support the findings of this study are not publicly available due to ethical and privacy restrictions but are available from the corresponding author upon reasonable request and with appropriate institutional approvals.

## Code availability

This study used the publicly available SuStaIn framework. All analyses were performed using the original publicly released SuStaIn code, which is available at: https://github.com/ucl-pond/pySuStaIn.
